# Physician self-reported treatment of brain metastases according to patients’ clinical and demographic factors and physician practice setting

**DOI:** 10.1186/1748-717X-7-188

**Published:** 2012-11-08

**Authors:** Marie-Adele S Kress, Naren Ramakrishna, Solomon B Makgoeng, Keith R Unger, Arnold L Potosky

**Affiliations:** 1 Department of Radiation Oncology, Georgetown University Medical Center, 3800 Reservoir Road, Washington, D.C, USA; 2 Department of Radiation Oncology, MD Anderson Cancer Center Orlando, Orlando, Florida, USA; 3 Cancer Control Program, Lombardi Comprehensive Cancer Center, Georgetown University Medical Center, Washington D.C, USA

**Keywords:** Brain metastases, Stereotactic radiosurgery, Whole brain radiation therapy, Treatment patterns, Physician survey

## Abstract

**Background:**

Limited data guide radiotherapy choices for patients with brain metastases. This survey aimed to identify patient, physician, and practice setting variables associated with reported preferences for different treatment techniques.

**Method:**

277 members of the American Society for Radiation Oncology (6% of surveyed physicians) completed a survey regarding treatment preferences for 21 hypothetical patients with brain metastases. Treatment choices included combinations of whole brain radiation therapy (WBRT), stereotactic radiosurgery (SRS), and surgery. Vignettes varied histology, extracranial disease status, Karnofsky Performance Status (KPS), presence of neurologic deficits, lesion size and number. Multivariate generalized estimating equation regression models were used to estimate odds ratios.

**Results:**

For a hypothetical patient with 3 lesions or 8 lesions, 21% and 91% of physicians, respectively, chose WBRT alone, compared with 1% selecting WBRT alone for a patient with 1 lesion. 51% chose WBRT alone for a patient with active extracranial disease or KPS=50%. 40% chose SRS alone for an 80 year-old patient with 1 lesion, compared to 29% for a 55 year-old patient. Multivariate modeling detailed factors associated with SRS use, including availability of SRS within one’s practice (OR 2.22, 95% CI 1.46-3.37).

**Conclusions:**

Poor prognostic factors, such as advanced age, poor performance status, or active extracranial disease, correspond with an increase in physicians’ reported preference for using WBRT. When controlling for clinical factors, equipment access was independently associated with choice of SRS. The large variability in preferences suggests that more information about the relative harms and benefits of these options is needed to guide decision-making.

## Background

Brain metastases are the most common intracranial tumor, occurring in 20-40% of cancer patients and accounting for 20% of cancer deaths annually [1]. Median survival is 1–2 months with corticosteroids alone [2] or six months with whole brain radiation therapy (WBRT) [3,4].

A major advance in the treatment of these patients was addition of surgery to WBRT for treatment of a single metastasis, which improved local control, distant intracranial control and neurologic survival compared to either modality alone [5,6]. A retrospective study demonstrated differential survival among patients undergoing WBRT according to recursive partitioning analysis (RPA) classes [7]; further prognostic refinements have incorporated histology and number of lesions [8].

More recently, stereotactic radiosurgery (SRS) has been used alone or with WBRT in patients with up to 4 metastases. When compared with WBRT alone, the addition of SRS has improved local control, functional autonomy and survival [5,9-11]. However, WBRT can have significant toxicities, including fatigue, drowsiness and suppressed appetite, and long-term difficulties with learning, memory, concentration, and depression [12-14]. The use of SRS alone controls limited disease and delays the time until WBRT is necessary for distant intracranial progression [12,15,16].

In most clinical trials of therapies for brain metastases, patients have been selected on the basis of having few metastases, stable extracranial disease, and excellent performance status. In clinical practice, patients with brain metastases are a heterogeneous population, and decision-making requires the synthesis of multiple variables.

The objective of this survey of radiation oncologists was to identify patient factors, physician characteristics, and practice setting variables associated with physicians’ preferred use of different techniques for treating brain metastases. This survey aimed to generate data that would allow physicians to: (1) compare their practice patterns to a national sample; (2) assess the influence of their practice environment on treatment choice; and (3) generate new hypotheses regarding appropriate treatment.

## Methods

This project was approved by the IRB of Harvard Medical School. The survey was launched online, and physician members of the American Society for Therapeutic Radiology and Oncology (ASTRO) were emailed a recruitment letter. Eligibility criteria included respondent status as a U.S. or Canadian physician in the ASTRO database, valid email address, and current management of patients with brain metastases, as reflected by the screener question. Respondents linked directly to the survey from the email, and there was no incentive for survey participation.

### Data collection

Data was de-identified and collected through the online survey tool for one month. We emailed surveys to 4357 physician members of ASTRO on September 26, 2008, and the survey was closed on October 26, 2008. 417 respondents answered at least one question, and 277 answered all demographic and clinical questions, for a response rate of 6%. Despite our low response rate, physician respondents were representative of practicing radiation oncologists when compared to respondents to the American College of Radiology’s (ACR) Survey of Radiation Oncologists. Our sample was similar to the ACR survey on selected characteristics such as sex (73% male in our survey, 77% in ACR), age (62% ages 35–54 in our survey, 65% in ACR) and being in private practice (52% in our survey, 48% in ACR) [17]. However, it was not possible to assess interest in SRS or palliative care, or use of advanced technology, among those included in the ACR sample, which limits the comparison.

**Figure 1:**
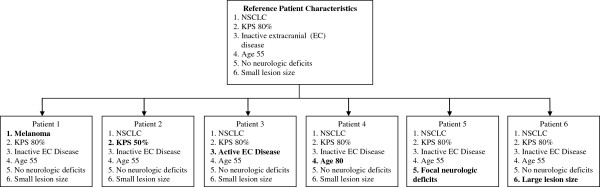
**Variations under assumptions of 1, 3, 8 metastases in each Reference Patient.** The survey sequentially varied the characteristics of each Reference Patient to create vignettes for Patients 1–6. The effect of each variation was evaluated under assumptions of 1, 3, and 8 lesions, respective to each vignette’s Reference Patient.

The survey was designed to: (1) describe radiation oncologists’ patterns of treatment of patients with brain metastases; and (2) identify clinical, demographic, and practice setting factors associated with treatment patterns. To test physician practices, a series of short hypothetical clinical vignettes were developed to assess respondents’ preferred treatment modalities. Vignettes have been demonstrated to be a valid study tool when compared with actual clinical practice patterns [18]. Treatment options for each vignette were identical: WBRT alone; WBRT with SRS; SRS alone; WBRT with surgery; or no treatment. We constructed 3 versions of a reference vignette: the first with 1 metastasis, the next with 3 metastases, and one with 8 metastases. Each reference vignette described a 55 year-old patient with non-small cell lung cancer, inactive extracranial disease, Karnofsky Performance Status (KPS) 80%, and asymptomatic, small brain lesion(s). For each of these 3 vignettes, we asked about 6 additional patients, modifying a single variable: melanoma histology, active extracranial disease, KPS 50%, presence of neurologic deficit, age of 80 years old, and large lesion (Figure 1).

Other survey items assessed factors related to the patient, physician, or practice setting. These questions included physician demographics, practice environment, availability of SRS, and opinions about the nature of intracranial disease and the toxicity of its treatment. A copy of our survey is included as supplementary material (Additional file 1: Appendix 1). Data regarding non-respondents were not collected.

### Statistical analysis

#### *Effects of patient clinical characteristics on treatment choices*

For the four category treatment choice responses (WBRT alone, WBRT with SRS, SRS alone, or surgery with WBRT), we used a series of multivariate binomial generalized estimating equation (GEE) models to estimate odds ratios that measured the effects of each change in patients’ clinical characteristics on the odds of each of 4 treatments choices relative to the odds of the remaining 3 alternatives. Since each vignette represented a repeat measurement on a physician, we considered treatment choices as correlated observations clustered within individual physicians. We used an exchangeable correlation structure to account for the correlation of physician responses between vignettes. Graphical techniques were used to assess model adequacy. We chose to use a series of binomial models to model a multi-category response because of the lack of available statistical software to implement multi-category GEE models with exchangeable correlation structure [19].

#### *Effects of patient* &*physician characteristics on odds of including SRS*

We grouped treatment responses that included SRS (SRS or WBRT with SRS) and compared them with the 3 remaining alternatives as a combined reference group (WBRT, WBRT with surgery, or no treatment) in a binomial GEE model that included patient clinical, physician and practice setting characteristics as covariates. These groupings were created to allow for exploration of factors contributing to integrating advanced technology (SRS) into the treatment plan, despite the fact that each treatment approach may have different clinical indications, as explored through the above-detailed models. Working correlations and clustering were treated as in the previous models.

All parameter estimates were tested for statistical significance at the 0.05 level. SAS® software version 9.2 was used in all analyses.

## Results

### Physician demographics and practice environment

The characteristics of our survey respondents are shown in Table 1. Sixty percent of respondents were in single-specialty group practices. Most practices were hospital-based, academic (38%) or private (30%). Seventy-six percent of respondents treated 10–50 patients with brain metastases per year. Forty-four percent of respondents performed SRS, while 35% had a colleague at their institution who performed SRS. Sixty-one percent of respondents had LINAC-based SRS, and 18% had no SRS equipment.

**Table 1:** Distribution of Physician Characteristics (N=277)

	**n**	**%**
**Total**		
No. of physicians with complete data	277	100
**Gender**		
Male	202	73
Female	75	27
**Race/ethnicity**		
Non-White	53	19
White	224	81
**Primary practice structure**		
Multi-specialty	111	40
Single	166	60
**Primary practice environment**		
Office-based, private	60	22
Office-based, academic	14	5
Hospital-based, private	84	30
Hospital-based, academic	105	38
Veterans'/military hospital	6	2
Other	8	3
**Years experience in specialty**		
Q1:0-5	70	25
Q2:6-14	77	28
Q3:15-20	61	22
Q4:21-40	69	25
**Approx. annual count of brain metastases patients**		
<10	18	7
10-50	210	76
>50	49	18
**Frequency of patient follow-up**		
At diagnosis only	3	1
Through active treatment for brain metastases	19	7
Through one post-treatment visit	95	34
Throughout the remaining course of their disease	152	55
Other	8	3

^1^ Respondents were permitted to select more than one modality.

^2^ Personal experience includes the respondent personally being treated for brain metastases, or having had a friend or family member treated for brain metastases.

* Stereotactic radiosurgery.

Physicians’ responses to the 21 vignettes varied substantially (Table 2). Multivariable modeling revealed clinical factors influencing treatment selection (Tables 3, 4, 5; complete results in Additional file 2: Appendix 2).

**Table 2:** Unadjusted Response (in %) Among Radiation Oncologist (N=277)

	**Lesions**											
	**1**				**3**				**8**			
	**Treatment Decisions (%)**				**Treatment Decisions (%)**				**Treatment Decisions (%)**			
	WBRT^*^	**WBRT+SRS**	**SRS†**	**SURG+WBRT**	**WBRT**	**WBRT+SRS**	**SRS**	**SURG+WBRT**	**WBRT**	**WBRT+SRS**	**SRS**	**SURG+WBRT**
**Patient Characteristics**												
1. Reference patient	1	26	29	44	21	54	23	2	91	7	3	0
2. Melanoma Metastasis	1	17	46	36	18	44	35	3	82	11	7	0
3. KPS 50	56	17	24	3	84	7	8	1	96	2	2	0
4. Extracranial Disease	51	19	23	7	73	15	11	0	94	3	2	0
5. Age 80	25	25	40	10	52	24	23	0	96	2	2	0
6. Focal Neurological Deficits	11	25	17	48	34	41	11	13	89	6	2	4
7. 3cm Brain Metastasis	11	22	5	63	42	29	2	27	72	16	0	11

^1^ Abbreviations as follows: Whole Brain Radiation Therapy (WBRT); Whole Brain Radiation Therapy with Stereotactic Radiosurgery (WBRT+SRS); Stereotactic Radiosurgery (SRS); Surgery with Whole Brain Radiation Therapy (SURG+WBRT).

^2^ The reference patient was a 55 year-old patient with non-small cell lung cancer, inactive extracranial disease, KPS 80%, and an asymptomatic, small brain lesion.

^3^ Patient characteristics were varied sequentially with each patient differing by a single characteristic from the reference patient as shown in Figure 1.

* Whole brain radiation therapy.

† Stereotactic radiosurgery.

**Table 3:** Odds Ratios for Choice of WBRT^* ^**alone versus SRS† Alone**

WBRT* vs. SRS	**OR (95%CI)** ††^1^	**P**
**Lesions**		
1 (referent)	1.00	
3	3.5 (2.7,4.4)	<.0001
8	59.8 (29.1,122.8)	<.0001
**Age**		
55 (referent)	1.00	
80	2.0 (1.5,2.6)	<.0001
**Extracranial Disease**		
None (referent)	1.00	
Active	5.5 (3.7,8.1)	<.0001
**Focal Neurological Deficits**		
Asymptomatic (referent)	1.00	
Symptomatic	2.7 (2.0,3.7)	<.0001
**Performance Status**§		
80 (referent)	1.00	
50	6.6 (4.4,9.9)	<.0001
**Lesion Size**		
Small (referent)	1.00	
Large	8.1 (5.3,12.4)	<.0001
**Melanoma**		
NSCLC¶ (referent)	1.00	
Melanoma	0.5 (0.4,0.6)	<.0001

**Table 4:** Odds Ratios for Choice of WBRT alone versus WBRT with SRS

**WBRT vs WBRT+SRS**	**OR (95%CI)**	**P**
**Lesions**		
1 (referent)	1.00	
3	2.5 (2.0,3.1)	<.0001
8	37.0 (24.6,55.5)	<.0001
**Age**		
55 (referent)	1.00	
80	4.6 (3.5,6.1)	<.0001
**Extracranial Disease**		
None (referent)	1.00	
Active	11.4 (8.2,15.9)	<.0001
**Focal Neurological Deficits**		
Asymptomatic (referent)	1.00	
Symptomatic	1.9 (1.5,2.3)	<.0001
**Performance Status**§		
80 (referent)	1.00	
50	18.0 (12.5,25.8)	<.0001
**Lesion Size**		
Small (referent)	1.00	
Large	1.9 (1.4,2.6)	<.0001
**Melanoma**		
NSCLC (referent)	1.00	
Melanoma	0.8 (0.7,1.0)	0.0698

**Table 5:** Odds Ratios for Choice of WBRT with SRS versus SRS alone

**WBRT+SRS vs SRS**	**OR (95%CI)**	**P**
**Lesions**		
1 (referent)	1.00	
3	2.1 (1.7,2.6)	<.0001
8	6.7 (2.4,18.5)	0.0002
**Age**		
55 (referent)	1.00	
80	0.6 (0.5,0.7)	<.0001
**Extracranial Disease**		
None (referent)	1.00	
Active	0.9 (0.7,1.3)	0.6576
**Focal Neurological Deficits**		
Asymptomatic (referent)	1.00	
Symptomatic	1.4 (1.1,1.7)	0.0021
**Performance Status**§		
80 (referent)	1.00	
50	0.7 (0.5,0.9)	0.0101
**Lesion Size**		
Small (referent)	1.00	
Large	5.6 (3.2,9.8)	<.0001
**Melanoma**		
NSCLC¶ (referent)	1.00	
Melanoma	0.5 (0.4,0.6)	<.0001

Notes

Odds ratios (OR) are quoted with their 95% confidence intervals in parentheses. "*" Denotes significant odds ratios at the 0.05 level.

The odds ratios compare odds of choosing each given treatment, with the odds of choosing the treatments serving as reference categories.

* Whole brain radiation therapy.

† Stereotactic radiosurgery.

†† Confidence intervals.

§ Karnofsky Performance Status.

¶ Non-Small Cell Lung Cancer.

### Whole brain radiation therapy alone

WBRT alone was selected frequently, particularly for patients with 8 metastases. For the 80 year-old patient with 3 or 8 metastases, WBRT was commonly preferred (52% and 96% vs. 21% and 91%, respectively, for the 55-year old patient, Table 2). Even for a patient with a single metastasis, 56% of respondents preferred WBRT alone if that patient had KPS 50%; 51% would choose WBRT if the patient had active extracranial disease. In adjusted analyses, all of the clinical variables (melanoma histology, KPS 50%, active extracranial disease, age of 80 years old, presence of focal neurologic deficits, and large lesion) were associated with a higher likelihood of respondents preferring WBRT alone versus either SRS alone (Table 3) or WBRT with SRS (Table 4), except for radioresistant histology.

### Addition of surgery

For the reference patient with a single metastasis, 44% of respondents selected surgery with WBRT, although most respondents selected a non-operative approach that included SRS (26% WBRT with SRS; 29% SRS alone, for a total of 55% of respondents). When the reference vignette was revised to include the presence of focal neurologic deficits, the distribution of responses was similar for those with 1 lesion, with 48% of respondents preferring surgery with WBRT. When considering patients with a single, large lesion, the percent of respondents choosing surgery with WBRT increased from 44% to 63%. After adjusting for all other clinical factors, respondents were more likely to choose surgery with WBRT rather than WBRT alone for patients with large versus smaller lesions (OR=1.9, 95% CI 1.3-2.8). For 3 or 8 lesions, age 80, active extracranial disease, and KPS 50%, respondents were more likely to choose WBRT alone than surgery with WBRT (Additional file 2: Appendix 2). Melanoma histology and presence of neurologic deficits did not correlate with respondents’ selections.

### Addition of stereotactic radiosurgery

SRS was commonly preferred by respondents for patients with 3 lesions (23% SRS alone; 54% SRS with WBRT, Table 2), and it largely replaced the use of surgery for the older patient with a single lesion (25% WBRT with SRS; 40% chose SRS alone). Presence of neurological deficits and large lesion size were associated with physicians’ preference for WBRT with SRS over SRS alone (Table 5). However, older age, poorer performance status and melanoma histology were associated with less frequent selection of WBRT with SRS versus SRS alone.

### Use of stereotactic radiosurgery

Multivariable analysis was performed to identify which factors were independently associated with including SRS as part of treatment (SRS or WBRT with SRS) compared to all other treatment choices (WBRT, WBRT with surgery, no treatment), adjusting for all other characteristics in Table 6. Number of metastases was strongly associated with treatment preferences: after adjustment for all other factors in the model, respondents were significantly more likely to favor SRS for 3 lesions than for 1 (OR=2.22, 95% CI 1.96-2.51), and physicians were 5 times less likely to choose an approach that included SRS for a patient with 8 lesions relative to patients with 1 lesion (OR=0.19, 95% CI 0.15-0.23).

**Table 6:** Results of logistic regression model showing the reported use of SRS^* ^**as part of treatment for brain metastases according to multiple clinical, sociodemographic, and practice setting factors**^**2**^

	**Including SRS**^**1**^	**p-value**
Lesions		
1 (reference)	1.00	
3	2.22 (1.96-2.51)	<.0001
8	0.19 (0.15-0.23)	<.0001
Karnofsky Performance Status		
80 (reference)	1.00	
50	0.38 (0.31-0.46)	<.0001
Tumor Characteristics		
Lung cancer (reference)	1.00	
Melanoma histology	2.84 (2.45-3.29)	<.0001
Extracranial disease		
No extracranial disease (reference)	1.00	
Active extracranial disease	0.56 (0.47-0.65)	<.0001
Age		
55 (reference)	1.00	
80	1.23 (1.07-1.41)	0.0034
Focal neurological deficits		
None (reference)	1.00	
Present	0.99 (0.85-1.14)	0.8492
Lesion size		
Small (reference)	1.00	
Large	0.58 (0.47-0.71)	<.0001
Race		
Other (reference)	1.00	
White	1.13 (0.83-1.52)	0.4415
Gender		
Male (reference)	1.00	
Female	1.07 (0.81-1.40)	0.6287
Specialization of Practice Setting		
Multispecialty (reference)	1.00	
Single	1.07 (0.82-1.39)	0.6036
Access to SRS		
None (reference)	1.00	
Personal use	3.57 (2.42-5.26)	<.0001
Available in practice	2.22 (1.46-3.37)	0.0002
WBRT † Adverse Effects Severity		
None (reference)	1.00	
At least minimal severity	0.55 (0.40-0.77)	0.0004
SRS Adverse Effects Severity		
None (reference)	1.00	
At least minimal severity	0.90 (0.53-1.51)	0.6804
Personal Experience with Brain Metastases		
Some personal (reference) ^3^	1.00	
Patients only	1.10 (0.81-1.50)	0.5369

^1^ Including SRS was defined as either use of SRS alone or with Whole Brain Radiation Therapy (WBRT).

^2^ The reference patient was a 55 year-old patient with non-small cell lung cancer, inactive extracranial disease, KPS 80%, and an asymptomatic, small brain lesion.

^3^ Personal experience includes the respondent personally being treated for brain metastases, or having had a friend or family member treated for brain metastases.

* Stereotactic radiosurgery.

† Whole brain radiation therapy.

Across all clinical vignettes, after adjusting for all other factors, poor KPS (OR=0.38, 95% CI 0.31-0.46), active extracranial disease (OR=0.56, 95% CI 0.47-0.65), and large lesion (OR=0.58, 95% CI 0.47-0.71) remained strongly negatively associated with the choice of SRS, while melanoma histology (OR=2.84, 95% CI 2.45-3.29) and advanced age (OR=1.23, 95% CI 1.07-1.41) were positively associated with choice of SRS. Physician access was the strongest factor associated with choosing SRS as part of treatment. Respondents with SRS capability in their own practice were more likely to favor its use for hypothetical patients than those without it (OR=2.22, 95% CI 1.46-3.37). As expected, those physicians who personally used SRS were more likely to recommend it than those who did not have it or use it personally in their practice (OR=3.57, 95% CI 2.42-5.26). Patient volume and physician seniority were examined, but were not associated with SRS use.

## Discussion

Treatment of patients with brain metastases is heterogeneous. WBRT is a standard therapy, with the addition of surgery or SRS to WBRT, or SRS used alone, reserved for selected patients on the basis of their clinical characteristics. One potential advantage of local therapy may be avoiding the toxicity of WBRT [12-14]. However, SRS, when used alone, has several disadvantages. SRS alone has been shown to be inferior to the combination of SRS with WBRT for durable local control and distant intracranial control [15]. When studying patients initially undergoing any local therapy – surgery or SRS – more patients required salvage if treated without WBRT [20]. Long-term cognitive outcomes have been shown to be more closely correlated with intracranial progression than with treatment modality, emphasizing the significance of intracranial control over short-term side effects [21,22].

Given the limited scope of current studies and the variability in outcomes, National Comprehensive Cancer Network (NCCN) guidelines allow for a wide range of treatment options including WBRT, surgical resection, or SRS, alone or in combinations [23]. Previous reviews of treatment patterns have demonstrated stable rates of surgery since the 1980s, with an increasing use of SRS [24]. Despite clinical trials limiting eligible patients to those with limited central nervous system disease, a recent survey demonstrated that more than half of physician respondents would consider using SRS as an initial treatment for patients with 5 or more intracranial lesions [25]. The increased utilization of SRS as well as the persistent heterogeneity in practice may be due to the time of dissemination of research into clinical practice, or the time to purchase and adoption of new technologies.

With mixed evidence and a heterogeneous patient population, treatment decision-making is complex. Significantly, our study demonstrates that although clinical factors, such as number of lesions and patient age, affected treatment selection, physician practice environment had a strong, independent effect on the use of SRS.

Factors related to the patient’s clinical condition affected treatment selection. There was increased use of WBRT for increasing number of lesions, which is consistent with the lack of evidence to support the use of local techniques for patients with numerous metastases. However, we observed that a substantial proportion of physicians still chose SRS as part of their approach for patients with multiple lesions, particularly for patients with 3 lesions. The increased use of SRS with 3 lesions as compared with 1 was possibly due to the use of surgery for a substantial proportion of patients with 1 lesion, and due to the use of SRS combined with WBRT in patients with 3 lesions. Interestingly, physicians overall selected WBRT for patients with 1, 3, or 8 lesions more often for patients who were frail (increased age, low KPS) and might suffer increased morbidity from WBRT. This finding was unexpected, since WBRT has been shown to cause side effects that might be difficult for frail patients with limited life expectancy to tolerate, such as increasing fatigue, worsening physical function, and deterioration of appetite [7,14,26]. Additional clinical factors may influence treatment selection, but were not addressed in this study, including tumor location and surgical accessibility; additional treatment options not evaluated include the use of SRS in combination with surgery, chemotherapy, and the role of hospice.

Practice environment and clinical expertise also influenced the use of SRS, even when controlling for clinical factors. Although practice type was not associated with the preference for SRS, the availability of SRS was significantly associated with its use, indicating that patients are more likely to receive this treatment if the physician they see practices it herself or has it available within her practice. This pattern of care could lead to under- or over-utilization of SRS: patients may have treatment guided more by a provider’s practice than by the patient’s clinical condition. Previous studies have demonstrated the association of physician specialization, board certification, treatment volume and time in practice with other cancer-related treatment decisions [27,28]. For example, diagnostic imaging use has increased when such imaging is performed at a self-referred facility [29]. Similarly, radiation oncologists may be prescribing complex treatment approaches more frequently when they have access to the facilities or equipment. Alternatively, this propensity for increased use of SRS with easy access may relate to physicians’ familiarity with their own clinical outcomes when using new technology. Our respondents may also have rates of access to SRS that are not comparable to those available nationwide, since the ACR survey did not report on the availability of SRS equipment.

Our study has several limitations due to its reliance on physician self-report as a proxy for practice, its timing, and the limited number of respondents. Clinical scenarios were hypothetical and treatment options were limited. Although physician surveys have shown a strong correlation between vignettes and actual practice [18], further objective validation of these data would be desirable, as the vignettes used in this survey were novel. Respondents to this survey were dominantly radiation oncologists, whose treatment decisions may be greatly impacted by other members of the inter-disciplinary oncology team not represented in this survey. Rates of radiosurgery utilization more than doubled between 2000 and 2005, so continued increases in the use of radiosurgery could have occurred since the completion of this survey [30]. Additional research has been published since 2008 that may have resulted in further shifts in practice patterns.

The limited number of respondents to our survey limits the generalizability of our findings. The response rate of 6% may indicate that the practice patterns outlined in this study are specific to a subgroup of clinicians with particular interest or expertise in radiosurgery and may not be indicative of global patterns of care. Although respondents were similar to those in the ACR survey, the comparison is limited due to the nature of the variables available; key issues, such as expertise with SRS or volume of patients brain metastases, were not available in the ACR survey for comparison. However, ours is the first study to document practice patterns using vignettes in this clinical setting.

## Conclusions

Although many patients with cancer develop brain metastases, there is little data to guide treatment decisions. Our study demonstrates the significant heterogeneity among radiation oncologists in general clinical practice even for patients with identical clinical characteristics. Certain non-clinical factors, such as access to SRS, appear to be key drivers of use of advanced technology. This finding raises the question about what additional incentives could be driving treatment selection in the absence of gold-standard evidence of the superiority of a single approach over other alternatives. Our findings from this survey also underscore the likely uncertainty or disagreement that may exist among radiation oncologists about the relative harms and benefits of different treatment approaches. This uncertainty is likely related to the lack of prospective randomized studies that compare specific single- and multi-modality approaches for the treatment of brain metastases. More research is needed that directly compares the effectiveness of these approaches for a variety of different clinical circumstances. It would also be important to investigate underlying non-clinical factors, such as physician environment, reimbursement, and technology access, which likely contribute to observed heterogeneity of care for patients with brain metastases.

## Abbreviations

WBRT: Whole brain radiation therapy; SRS: Stereotactic radiosurgery; KPS: Karnofsky performance status; RPA: Recursive partitioning analysis; ASTRO: American society for therapeutic radiation oncology; ACR: American college of radiology; GEE: Generalized estimating equation; NCCN: National comprehensive cancer network.

## Competing interests

Dr. Ramakrishna has received speaker’s honoraria from and prepared educational materials for Brainlab Ag, Heimstetten, Germany. The remaining authors have no conflicts of interest to disclose.

## Authors’ contributions

NR and MK conceived of the study, designed the survey, and completed data collection. MK, KU, SM, and AP performed statistical analysis and data interpretation. MK, SM, and AP drafted the manuscript. All authors read and approved the final manuscript.

## Supplementary Material

Additional file 1 **Appendix 1.** Complete physician survey. Click here for file

Additional file 2 **Appendix 2.** Odds Ratios and Confidence Intervals Comparing the Odds of Treatment Choices for Different Patient Characteristics. Click here for file
