# Supplementary material for: Physician self-reported treatment of brain metastases according to patients’ clinical and demographic factors and physician practice setting
Source: Radiat Oncol. 2012 Nov 8;7:188. doi: 10.1186/1748-717X-7-188 (PMC3533820; doi:10.1186/1748-717X-7-188)
Supplement: Additional file 1 — Appendix 1. Complete physician survey. [file 1748-717X-7-188-S1.pdf]

Physician Practices in the Treatment of Brain Metastases

Introduction.

Thank you for your interest to participate in our survey.

The management of patients with brain metastases remains controversial with a broad range of patterns of practice. Our survey aims to explore physician practice patterns for the treatment of patients with brain metastases as well as examine multiple factors that may influence physician decision-making.

This survey is anonymous and brief; please feel free to email Marie-Adele_Sorel@hms.harvard.edu with any questions.

*** 1. Have you, in the past year, been involved in the decision-making for care of patients with brain metastases?**

- Yes
- No

If you answered "No" to this question, please do not complete the rest of this survey. If you answered "Yes," please continue to the next page.

Physician Practices in the Treatment of Brain Metastases

Patient case #1: Mr. A.

Following are three clinical vignettes: one patient with one brain metastasis, another with three brain metastases, and a final patient with eight brain metastases. For each of these cases and the variations that follow, please select the answer choice that best represents your initial management plan for that patient.

Questions 2-8 below refer to the following patient, Mr. A, with a solitary brain metastasis:
Mr. A, an asymptomatic 55 year-old man with non-small cell lung cancer and inactive extracranial disease presents with KPS of 80. Routine restaging studies reveal interval finding of a solitary, 1-cm enhancing lesion, consistent in appearance with a brain metastasis from his lung cancer.

*** 2. Assuming that the lesion is a brain metastasis from his lung cancer, what would be your initial management plan for this patient?**

- Whole brain radiation therapy alone
- Whole brain radiation therapy with stereotactic radiosurgery boost
- Stereotactic radiosurgery alone
- Surgery with whole brain radiation therapy
- No treatment

*** 3. Referring back to Mr. A's initial case, if he instead had a KPS of 50, what would be your initial management plan?**

- Whole brain radiation therapy alone
- Whole brain radiation therapy with stereotactic radiosurgery boost
- Stereotactic radiosurgery alone
- Surgery with whole brain radiation therapy
- No treatment

*** 4. Referring back to Mr. A's initial case, if his brain metastasis instead was from melanoma, what would be your initial management plan?**

- Whole brain radiation therapy alone
- Whole brain radiation therapy with stereotactic radiosurgery boost
- Stereotactic radiosurgery alone
- Surgery with whole brain radiation therapy
- No treatment

*** 5. Referring back to Mr. A's initial case, if he instead had active extracranial disease, what would be your initial management plan?**

- Whole brain radiation therapy alone
- Whole brain radiation therapy with stereotactic radiosurgery boost
- Stereotactic radiosurgery alone
- Surgery with whole brain radiation therapy
- No treatment

*** 6. Referring back to Mr. A's initial case, if he instead was 80 years old, what would be your initial management plan?**

- Whole brain radiation therapy alone
- Whole brain radiation therapy with stereotactic radiosurgery boost
- Stereotactic radiosurgery alone
- Surgery with whole brain radiation therapy
- No treatment

*** 7. Referring back to Mr. A's initial case, if he instead did have focal neurological deficits related to his brain metastasis, what would be your initial management plan?**

- Whole brain radiation therapy alone
- Whole brain radiation therapy with stereotactic radiosurgery boost
- Stereotactic radiosurgery alone
- Surgery with whole brain radiation therapy
- No treatment

*** 8. Referring back to Mr. A's initial case, if he instead had a 3-cm brain metastasis with associated edema and related focal neurological deficits, what would be your initial management plan?**

- Whole brain radiation therapy alone
- Whole brain radiation therapy with stereotactic radiosurgery boost
- Stereotactic radiosurgery alone
- Surgery with whole brain radiation therapy
- No treatment

Patient case #2: Mr. B.

Questions 9-15 below refer to the following patient, Mr. B, with three brain metastases:
Mr. B, an asymptomatic 55 year-old man with non-small cell lung cancer and inactive extracranial disease presents with KPS of 80. Routine restaging studies reveal interval finding of three 1-cm enhancing lesions consistent in appearance with brain metastases from his lung cancer.

*** 9. Assuming that the lesions are brain metastases, what would be your initial management plan for this patient?**

- Whole brain radiation therapy alone
- Whole brain radiation therapy with stereotactic radiosurgery boost
- Stereotactic radiosurgery alone
- Surgery with whole brain radiation therapy
- No treatment

*** 10. Referring back to Mr. B's initial case, if he instead had a KPS of 50, what would be your initial management plan?**

- Whole brain radiation therapy alone
- Whole brain radiation therapy with stereotactic radiosurgery boost
- Stereotactic radiosurgery alone
- Surgery with whole brain radiation therapy
- No treatment

*** 11. Referring back to Mr. B's initial case, if his brain metastases instead were from melanoma, what would be your initial management plan?**

- Whole brain radiation therapy alone
- Whole brain radiation therapy with stereotactic radiosurgery boost
- Stereotactic radiosurgery alone
- Surgery with whole brain radiation therapy
- No treatment

*** 12. Referring back to Mr. B's initial case, if he instead had active extracranial disease, what would be your initial management plan?**

- Whole brain radiation therapy alone
- Whole brain radiation therapy with stereotactic radiosurgery boost
- Stereotactic radiosurgery alone
- Surgery with whole brain radiation therapy
- No treatment

*** 13. Referring back to Mr. B's initial case, if he instead was 80 years old, what would be your initial management plan?**

- Whole brain radiation therapy alone
- Whole brain radiation therapy with stereotactic radiosurgery boost
- Stereotactic radiosurgery alone
- Surgery with whole brain radiation therapy
- No treatment

*** 14. Referring back to Mr. B's initial case, if he instead had focal neurological deficits related to his brain metastases, what would be your initial management plan?**

- Whole brain radiation therapy alone
- Whole brain radiation therapy with stereotactic radiosurgery boost
- Stereotactic radiosurgery alone
- Surgery with whole brain radiation therapy
- No treatment

*** 15. Referring back to Mr. B's initial case, if the largest of his brain metastases was 4-cm, what would be your initial management plan?**

- Whole brain radiation therapy alone
- Whole brain radiation therapy with stereotactic radiosurgery boost
- Stereotactic radiosurgery alone
- Surgery with whole brain radiation therapy
- No treatment

Patient case #3: Mr. C.

Questions 16-22 below refer to the following patient, Mr. C, with eight brain metastases:
Mr. C, an asymptomatic 55 year-old man with non-small cell lung cancer and inactive extracranial disease presents with KPS of 80. Routine restaging studies reveal interval finding of eight enhancing lesions, 5-10 mm in size, consistent in appearance with brain metastases from his lung cancer.

*** 16. Assuming that the lesions are brain metastases, what would be your initial management plan for this patient?**

- Whole brain radiation therapy alone
- Whole brain radiation therapy with stereotactic radiosurgery boost
- Stereotactic radiosurgery alone
- Surgery with whole brain radiation therapy
- No treatment

*** 17. Referring back to Mr. C's initial case, if he instead had a KPS of 50, what would be your initial management plan?**

- Whole brain radiation therapy alone
- Whole brain radiation therapy with stereotactic radiosurgery boost
- Stereotactic radiosurgery alone
- Surgery with whole brain radiation therapy
- No treatment

*** 18. Referring back to Mr. C's initial case, if his brain metastases instead were from melanoma, what would be your initial management plan?**

- Whole brain radiation therapy alone
- Whole brain radiation therapy with stereotactic radiosurgery boost
- Stereotactic radiosurgery alone
- Surgery with whole brain radiation therapy
- No treatment

*** 19. Referring back to Mr. C's initial case, if he instead had active extracranial disease, what would be your initial management plan?**

- Whole brain radiation therapy alone
- Whole brain radiation therapy with stereotactic radiosurgery boost
- Stereotactic radiosurgery alone
- Surgery with whole brain radiation therapy
- No treatment

*** 20. Referring back to Mr. C's initial case, if he instead was 80 years old, what would be your initial management plan?**

- Whole brain radiation therapy alone
- Whole brain radiation therapy with stereotactic radiosurgery boost
- Stereotactic radiosurgery alone
- Surgery with whole brain radiation therapy
- No treatment

*** 21. Referring back to Mr. C's initial case, if he instead had focal neurological deficits related to his brain metastases, what would be your initial management plan?**

- Whole brain radiation therapy alone
- Whole brain radiation therapy with stereotactic radiosurgery boost
- Stereotactic radiosurgery alone
- Surgery with whole brain radiation therapy
- No treatment

*** 22. Referring back to Mr. C's initial case, if the largest of his brain metastases was 4-cm, what would be your initial management plan?**

- Whole brain radiation therapy alone
- Whole brain radiation therapy with stereotactic radiosurgery boost
- Stereotactic radiosurgery alone
- Surgery with whole brain radiation therapy
- No treatment

Decision-making.

* **23. Please select the answer for each of the following factors that best describes how you (the physician) think that the factor influences your decision-making of how to manage patients with brain metastases.**

	No influence on my decision-making	Minimal influence	Moderate influence	Strong influence
Patient's concern about cognitive decline.				
Patient's desire to maximize quality of life.				
Logistical convenience of treatment for patient.				
Extent of patient's symptoms from intracranial disease.				
Patient's desire for minimal intervention.				
Patient currently enrolled in hospice.				
Patient's desire for aggressive management.				
Waiting time until treatment is available.				
Financial cost of treatment.				
Patient's insurance plan or lack thereof.				

Please answer the following questions regarding your understanding of disease that is metastatic to the brain and its treatment.

* **24. At what number of metastases would you consider disease to be diffuse and not amenable to local treatment? Please enter a whole number.**

* **25. In patients with known metastatic disease who are asymptomatic, which of the following do you recommend for screening for brain metastases?**

- No screening
- One screening at the time of initial diagnosis
- Screening once per year
- Screening more than once per year

*** 26. How do you describe, to your patients, the severity of fatigue related to whole brain radiation therapy (WBRT)?**

- None
- Minimal
- Mild
- Moderate
- Severe

*** 27. How do you describe, to your patients, the severity of decline in quality of life related to WBRT?**

- None
- Minimal
- Mild
- Moderate
- Severe

*** 28. How do you describe, to your patients, the severity of cognitive decline related to WBRT?**

- None
- Minimal
- Mild
- Moderate
- Severe

*** 29. How do you describe, to your patients, the severity of radiation necrosis related to stereotactice radiosurgery (SRS)?**

- None
- Minimal
- Mild
- Moderate
- Severe

*** 30. Are you more or less likely to use SRS than you were one year ago?**

- Much more likely
- Somewhat more likely
- No difference
- Somewhat less likely
- Much less likely

*** 31. Than you were five years ago?**

- Much more likely
- Somewhat more likely
- No difference
- Somewhat less likely
- Much less likely

*** 32. Than you were 10 years ago?**

- Much more likely
- Somewhat more likely
- No difference
- Somewhat less likely
- Much less likely

Demographics.

Please answer the following questions about yourself and your medical and/or surgical practice.

*** 33. What is your primary specialty of practice?**

- Medical oncology
- Radiation oncology
- Surgical oncology
- Neuro-oncology
- Neurosurgery
- Other

(please specify)

*** 34. Please indicate the number of years for which you have practiced in your primary specialty.**

*** 35. Please enter the year in which you were born.**

*** 36. Please select your gender.**

- Male
- Female
- Other

*** 37. Please select your race(s)/ethnicit(y/ies).**

- White
- Black, African American
- American Indian or Alaska Native
- Asian Indian
- Chinese
- Filipino
- Japanese
- Korean
- Vietnamese
- Native Hawaiian
- Other

(please specify)

*** 38. Please enter the 5-digit zip code in which you currently practice.**

*** 39. Which of the following best describes the primary practice in which you work?**

- Solo practice
- Group practice - single specialty
- Group practice - multiple specialty
- Other

(please specify)

*** 40. Which of the following best describes your primary practice environment?**

- Office-based, private
- Office-based, academic
- Hospital-based, private
- Hospital-based, academic
- Veterans'/military hospital
- Other

(please specify)

*** 41. Please rank the following in terms of the proportion of time you spend on each, with "1" designating the area in which you spend most of your time, and "4" designating the area in which you spend the least amount of time.**

	Most time (1)	Significant time (2)	Little time (3)	Least time (4)
Clinical care				
Research				
Teaching				
Administration				

*** 42. For how many patients with brain metastases do you care each year?**

- <10 patients
- 10-50 patients
- >50 patients

*** 43. How often do you follow-up with patients after their diagnosis of brain metastases?**

- At diagnosis only
- Through active treatment for brain metastases
- Through one post-treatment visit
- Throughout the remaining course of their disease
- Other

(please specify)

*** 44. Please answer the following about the availability of stereotactic radiosurgery (SRS):**

- I perform SRS.
- I do not perform SRS, but a colleague in my practice and/or institution does.
- I do not perform SRS, nor does anyone in my practice or institution, but a colleague in my city does.
- I do not perform SRS, nor does anyone in my practice, institution, or city.

*** 45. Which of the following personal experiences have you had with treatment of brain metastases? (Select all that apply)**

- I have been treated for brain metastases.
- A family member of mine has been treated for brain metastases.
- A friend of mine has been treated for brain metastases.
- I know no one, apart from my patients, who has been treated for brain metastases.

*** 46. Which of the following types of radiosurgery equipment do you have at your place of practice for the treatment of brain metastases? (Select all that apply)**

- Gamma Knife
- LINAC SRS
- Cyberknife
- None
- Other

(please specify)

Physician Practices in the Treatment of Brain Metastases

Thank you.

Thank you for taking the time to complete our survey.

47. Please feel free to enter any comments about this survey below.
