# Supplementary material for: Physician self-reported treatment of brain metastases according to patients’ clinical and demographic factors and physician practice setting
Source: Radiat Oncol. 2012 Nov 8;7:188. doi: 10.1186/1748-717X-7-188 (PMC3533820; doi:10.1186/1748-717X-7-188)
Supplement: Additional file 2 — Appendix 2. Odds Ratios and Confidence Intervals Comparing the Odds of Treatment Choices for Different Patient Characteristics. [file 1748-717X-7-188-S2.docx]

Appendix 2. Odds Ratios and Confidence Intervals Comparing the Odds of Treatment Choices for Different Patient Characteristics.

|  |  |  |  |  |  |  |  |  |  |
| --- | --- | --- | --- | --- | --- | --- | --- | --- | --- |
|  |  |  | **Referent Treatment^3^** | | | | | | |
| **Treatments^2^ and Patient Characteristics** | |  | **WBRT**^*^ |  | **WBRT+SRS**† |  | **SRS** |  | **WBRT + Surgery** |
|  |  |  | **TBL** |  | **TBL 2** |  | **TBL 3** |  | **TBL 4** |
| **WBRT** | |  |  |  |  |  |  |  |  |
|  | 3 Lesions (vs 1 lesion) |  |  |  | 2.5 (2.0,3.1)* |  | 3.5 (2.7,4.4)* |  | 20.0 (14.3,27.9)* |
|  | 8 Lesions (vs 1 lesion) |  |  |  | 37.0 (24.6,55.5)* |  | 59.8 (29.1,122.8)* |  | 152.6 (99.7,233.4)* |
|  | Age 80 (vs age 55) |  |  |  | 4.6 (3.5,6.1)* |  | 2.0 (1.5,2.6)* |  | 13.5 (8.9,20.2)* |
|  | Active extracranial disease (vs inactive) |  |  |  | 11.4 (8.2,15.9)* |  | 5.5 (3.7,8.1)* |  | 35.9 (23.0,56.2)* |
|  | Neurological deficits (vs asymptomatic) |  |  |  | 1.9 (1.5,2.3)* |  | 2.7 (2.0,3.7)* |  | 1.0 (0.7,1.4) |
|  | Performance Status (KPS†† = 50 vs 80) |  |  |  | 18.0 (12.5,25.8)* |  | 6.6 (4.4,9.9)* |  | 86.4 (45.5,163.7)* |
|  | Large lesion size (vs small) |  |  |  | 1.9 (1.4,2.6)* |  | 8.1 (5.3,12.4)* |  | 0.5 (0.4,0.8)* |
|  | Melanoma (vs NSCLC\|\|) |  |  |  | 0.8 (0.7,1.0) |  | 0.5 (0.4,0.6)* |  | 1.0 (0.8,1.4) |
| **WBRT + SRS** | |  |  |  |  |  |  |  |  |
|  | 3 Lesions (vs 1 lesion) |  | 0.4 (0.3,0.5)* |  |  |  | 2.1 (1.7,2.6)* |  | 8.0 (6.0,10.8)* |
|  | 8 Lesions (vs 1 lesion) |  | 0.0 (0.0,0.0)* |  |  |  | 6.7 (2.4,18.5)* |  | 6.6 (4.5,9.8)* |
|  | Age 80 (vs age 55) |  | 0.2 (0.2,0.3)* |  |  |  | 0.6 (0.5,0.7)* |  | 3.4 (2.3,5.2)* |
|  | Active extracranial disease (vs inactive) |  | 0.1 (0.1,0.1)* |  |  |  | 0.9 (0.7,1.3) |  | 4.0 (2.4,6.5)* |
|  | Neurological deficits (vs asymptomatic) |  | 0.5 (0.4,0.7)* |  |  |  | 1.4 (1.1,1.7)* |  | 0.6 (0.4,0.8)* |
|  | Performance Status (KPS = 50 vs 80) |  | 0.1 (0.0,0.1)* |  |  |  | 0.7 (0.5,0.9)* |  | 7.7 (3.7,16.1)* |
|  | Large lesion size (vs small) |  | 0.5 (0.4,0.7)* |  |  |  | 5.6 (3.2,9.8)* |  | 0.3 (0.2,0.4)* |
|  | Melanoma (vs NSCLC) |  | 1.2 (1.0,1.5) |  |  |  | 0.5 (0.4,0.6)* |  | 1.0 (0.8,1.2) |
| **SRS** | |  |  |  |  |  |  |  |  |
|  | 3 Lesions (vs 1 lesion) |  | 0.3 (0.2,0.4)* |  | 0.5 (0.4,0.6)* |  |  |  | 2.0 (1.6,2.5)* |
|  | 8 Lesions (vs 1 lesion) |  | 0.0 (0.0,0.0)* |  | 0.1 (0.1,0.4)* |  |  |  | 1.0 (0.7,1.4) |
|  | Age 80 (vs age 55) |  | 0.5 (0.4,0.7)* |  | 1.7 (1.4,2.1)* |  |  |  | 2.4 (1.9,3.1)* |
|  | Active extracranial disease (vs inactive) |  | 0.2 (0.1,0.3)* |  | 1.1 (0.8,1.4) |  |  |  | 1.9 (1.5,2.5)* |
|  | Neurological deficits (vs asymptomatic) |  | 0.4 (0.3,0.5)* |  | 0.7 (0.6,0.9)* |  |  |  | 0.4 (0.3,0.5)* |
|  | Performance Status (KPS = 50 vs 80) |  | 0.2 (0.1,0.2)* |  | 1.5 (1.1,2.1)* |  |  |  | 2.7 (1.9,4.0)* |
|  | Large lesion size (vs small) |  | 0.1 (0.1,0.2)* |  | 0.2 (0.1,0.3)* |  |  |  | 0.1 (0.0,0.1)* |
|  | Melanoma (vs NSCLC) |  | 1.9 (1.5,2.4)* |  | 2.1 (1.7,2.7)* |  |  |  | 1.6 (1.3,2.0)* |
| **WBRT + Surgery** | |  |  |  |  |  |  |  |  |
|  | 3 Lesions (vs 1 lesion) |  | 0.1 (0.0,0.1)* |  | 0.1 (0.1,0.2)* |  | 0.5 (0.4,0.6)* |  |  |
|  | 8 Lesions (vs 1 lesion) |  | 0.0 (0.0,0.0)* |  | 0.2 (0.1,0.2)* |  | 1.0 (0.7,1.5) |  |  |
|  | Age 80 (vs age 55) |  | 0.1 (0.0,0.1)* |  | 0.3 (0.2,0.4)* |  | 0.4 (0.3,0.5)* |  |  |
|  | Active extracranial disease (vs inactive) |  | 0.0 (0.0,0.0)* |  | 0.3 (0.2,0.4)* |  | 0.5 (0.4,0.7)* |  |  |
|  | Neurological deficits (vs asymptomatic) |  | 1.0 (0.7,1.4) |  | 1.7 (1.3,2.3)* |  | 2.7 (2.0,3.7)* |  |  |
|  | Performance Status (KPS = 50 vs 80) |  | 0.0 (0.0,0.0)* |  | 0.1 (0.1,0.3)* |  | 0.4 (0.2,0.5)* |  |  |
|  | Large lesion size (vs small) |  | 1.9 (1.3,2.8)* |  | 3.4 (2.5,4.7)* |  | 18.5 (8.7,39.5)* |  |  |
|  | Melanoma (vs NSCLC) |  | 1.0 (0.7,1.3) |  | 1.0 (0.8,1.3) |  | 0.6 (0.5,0.8)* |  |  |
| **Notes:** | |  |  |  |  |  |  |  |  |
| ^1^ | Odds ratios are quoted with their confidence intervals in brackets. "*" Denotes significant odds ratios at the 0.05 level. | | | | | | | | |
| ^2^ | Each scenario represents a single departure from the characteristics of a baseline scenario, which was a patient, 55 years-old with non-small cell lung cancer, inactive extracranial disease, KPS 80%, and a single asymptomatic, small brain lesion. | | | | | | | | |
| ^3^ | The odds ratios compare odds of choosing each given treatment, with the odds of choosing the treatments serving as reference/comparison treatments. | | | | | | | | |
|  | *Abbreviations:* Whole Brain Radiation Therapy (WBRT); Stereotactic Radiosurgery (SRS); Non-small cell lung cancer (NSCLC). | | | | | | | | |

_______________________________________

* Whole brain radiation therapy

† Stereotactic radiosurgery

†† Karnofsky Performance Status

|| Non-Small Cell Lung Cancer
